# Myosin XI motors: back on the scene at the division machine

**DOI:** 10.1093/jxb/ery143

**Published:** 2018-05-25

**Authors:** Henrik Buschmann

**Affiliations:** Botany Department, School of Biology and Chemistry, University of Osnabrück, Barbarastrasse, Osnabrück, Germany

**Keywords:** Arabidopsis, cell division, microtubules, MyoB, myosin XI, polar auxin transport, root organogenesis

## Abstract

This article comments on:

**Abu-Abied M, Belausov E, Hagay S, Peremyslov V, Dolja V, Sadot E.** 2018. Myosin XI-K is involved in root organogenesis, polar auxin transport and cell division. Journal of Experimental Botany **69**, 2869–2881.

**Plant myosin XI motors are renowned for their function in cytoplasmic streaming. But the cell biology of myosin XI has taken an unexpected turn as a study by** **Abu-Abied *et al.* (2018)** **suggests a role of the motor in auxin transport and cell division plane alignment.**

Only two types of myosins are present in green algae and land plants, myosin VIII and myosin XI, but in land plants each type is represented by a small gene family (Nebenfuhr and Dixit, 2018). Strictly speaking, there is one more myosin-like sequence present in the genomes of plants, the chimeric motor KCBP, which sports an N-terminal MyTH4-FERM domain with significant BLAST homology to myosin VII. However, the motor domain of KCBP is a 14-type kinesin (Reddy and Day, 2000). While a function of myosin VIII in cell division has been reported before, myosin XI is probably best known for its impact on cytoplasmic streaming (Tominaga *et al.*, 2013). Indeed, some myosin XI motors are the fastest known cytoskeletal motors, and these are responsible for the remarkably vigorous cyclosis observed in *Chara* and related algae (Higashi-Fujime *et al.*, 1995).

Recent research has revealed details of myosin XI-mediated motility in plants (Sparkes *et al.*, 2008; Avisar *et al.*, 2009), and it was found that specific myosin receptors, so-called MyoB proteins, are involved in myosin attachment to certain cargo (Peremyslov *et al.*, 2013). Previous research has also used mutants to pinpoint the molecular functions of myosin XI. Knocking out myosin XI in Arabidopsis (frequently using a ‘3KO’ triple mutant of a group of closely related myosin XI genes) produced several phenotypes: trichomes, root hairs and pavement cells all showed defects in cellular differentiation (Peremyslov *et al.*, 2010; Ojangu *et al.*, 2012; Duan and Tominaga, 2018).

## Myosin XI 3KO mutants have auxin-related and cell division phenotypes

The new paper by Abu-Abied *et al.* (2018) goes much further, reporting on analyses of the root growth of myosin 3KO plants in detail. Surprisingly, they found that the plants show several defects that point to the phytohormone auxin: myosin 3KO plants have surplus lateral roots, they mislocalize the PIN1 auxin exporters of stele cells and they show an aberrant auxin gradient in the root. But the authors found additional phenotypes: myosin 3KO plants show striking division-plane orientation defects in the stele, milder defects of the same type in endodermal/epidermal cells of the root meristem, and an increase in the time cells spend in mitosis and cytokinesis. There had been little evidence that myosin XI is involved in auxin signalling and cell division, and so the paper contributes a significant step forward in our understanding of myosin XI function in vascular plants.

As exciting as these novel phenotypes may be, it is not that easy to put them into a simple coherent model. Are these phenotypes interrelated, and if yes, what is cause and what is effect? As humans we tend to infer causal relationships where sometimes there are just correlations. When speaking to my students I ask them initially to view the different phenotypes presented by a given mutant as entirely independent. I suggest comparing a set of phenotypes with the spokes of a wheel (see Box 1): *a priori* we know only about one causal relationship, which is the lack of a protein as the basis for all the separate phenotypes observed (here: the myosin XI motor). The establishment of further causal relationships needs careful experimental testing. Coming back to the myosin 3KO phenotypes presented by Abu-Abied and colleagues: could it be that the auxin effects are the basis for the cell division defects? Or is it rather the other way around?

**Box 1. Phenotypic wheel for the *Arabidopsis myosin 3KO* mutant**

The *myosin 3KO* mutant has many described phenotypes, as indicated on the spokes of the wheel (though not all phenotypes are depicted). It is tempting to speculate about causal relationships between these phenotypes, though this depiction emphasizes that we should initially view the different phenotypes presented by a given mutant as independent. However, one speculative interaction is indicated by the arrow.

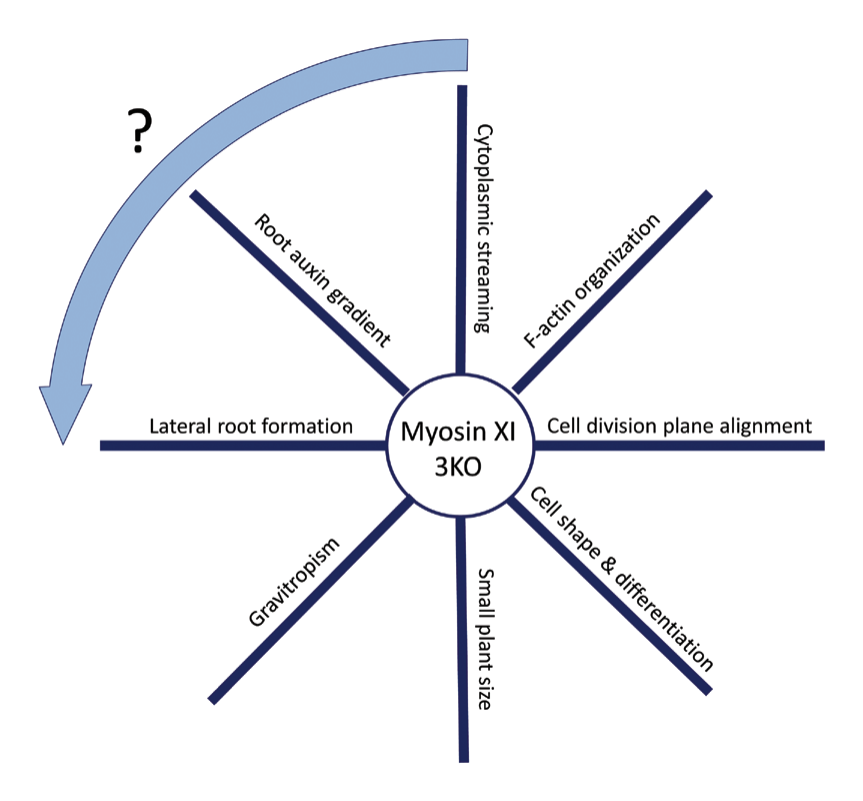

While this is a tricky question, Abu-Abied *et al.* provide some evidence that myosin XI has a genuine function in cell division, suggesting that the cell division phenotypes observed may be independent from the defect in auxin signalling. Plant cell division involves the succession of three microtubule arrays, the preprophase band, the mitotic spindle and the cytokinetic phragmoplast. The phragmoplast expands centrifugally and assembles in its midline the cell plate, the nascent division wall. In wild-type cells, the phragmoplast and cell plate grow back to the position on the parental membrane that was occupied and marked by the preprophase band before mitosis (Lloyd and Buschmann, 2007). By following a complemented 3KO mutant expressing YFP coupled to a myosin XI isotype through cell division, Abu-Abied *et al.* found that the motor associates with early cell plates and with the rim of the expanding cell plate during late cytokinesis. Interestingly, myosin XI of Arabidopsis is also found at the parental membrane for some time during pro-metaphase and then again during cytokinesis (Box 2). This membrane region is also known as the cortical division zone and is occupied by the preprophase band before nuclear envelope breakdown (Smertenko *et al.*, 2017). Given that the myosin 3KO mutant has defects in division plane orientation, the localization pattern described may be taken as support for the idea that myosin XI has a function in cell plate guidance. In another recent paper, Sun *et al.* (2018) observed a similar localization of a myosin XI paralogue to cell plates of the moss *Physcomitrella*.

**Box 2. Dividing plant cell and myosin XI localization**

The diagram shows a cell at cytokinesis with the phragmoplast microtubules and cell plate highlighted. Myosin XI (green) localizes to the parental plasma membrane (the cortical division zone) and to the edge of the cell plate.

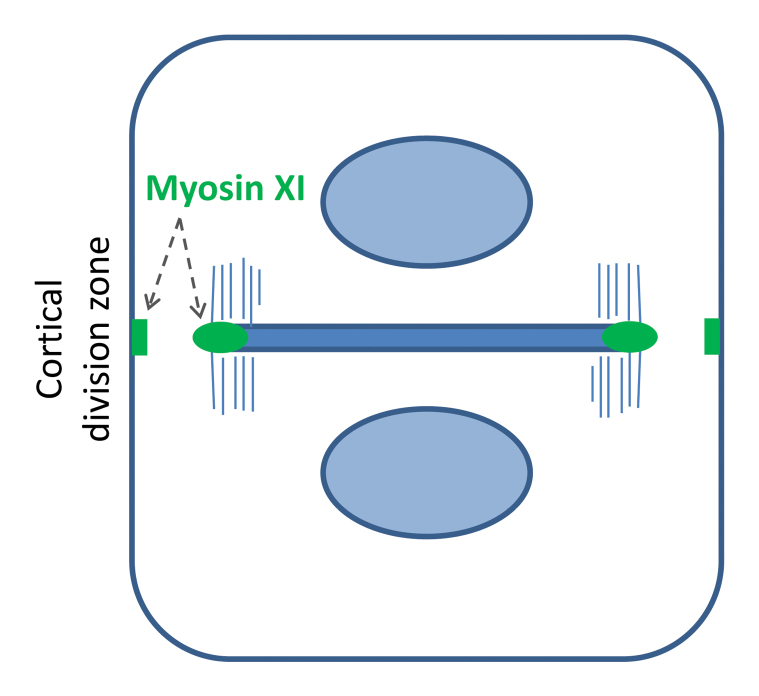

## Have you seen the bridge?

Research on the mechanism of cell division plane orientation and, specifically, cell plate guidance has produced a set of novel molecular players in recent years. Most of the proteins discovered point directly towards involvement of the microtubule cytoskeleton, perhaps because the respective plant mutants have strong phenotypes. However, imaging and inhibitor studies have previously shown that the actin cytoskeleton must have an important role in the process of cell plate guidance (Traas *et al.*, 1987; Sano *et al.*, 2005). It is therefore safe to assume that the ‘bridge’ connecting the phragmoplast and cell plate with the plasma membrane area of the cortical division zone should be made of both F-actin and microtubules.

But the puzzle is incomplete as we know of only a handful of proteins involved and the mechanisms suggested to explain cell plate guidance are diverse (Lipka *et al.*, 2014; Wu and Bezanilla, 2014; Buschmann *et al.*, 2015). Importantly, recent reports suggest that myosins and a myosin-like sequence are involved. Myosin VIII, which was found to be present in the cortical division zone and on the phragmoplast of *Physcomitrella* was shown to interact with both F-actin and microtubules. Knockout mutants of myosin VIII showed division-plane orientation defects in the protonema of *Physcomitrella* (Wu and Bezanilla, 2014). Interestingly, the chimeric motor KCBP, which contains the myosin-like MyTH4-FERM domain in its N-terminus, is also found in the cortical division zone of dividing tobacco and Arabidopsis cells (Buschmann *et al.*, 2015). The paper by Abu-Abied *et al.* now shows that myosin XI of Arabidopsis associates with the cortical division zone and with the expanding cell plate. Because the 3KO mutants of myosin XI in Arabidopsis show skewed division planes in the stele the analyses provide important molecular genetic evidence that myosin is involved in the division plane alignment of higher plants.

## On new tracks

Together, the new results show that the cortical division zone of plants contains a group of myosins and myosin-like sequences. This alone is curious, as the cortical division zone itself is thought be F-actin deficient (Cleary, 1995; Sano *et al.*, 2005). Non-plant eukaryotes divide by constriction using a contractile ring containing actin and myosin, while plants divide centrifugally using a phragmoplast with associated cell plate (Rasmussen *et al.*, 2011; Cheffings *et al.*, 2016). Perhaps the enigmatic cortical division zone of plants is simply a modified actomyosin ring without actin. The recent discovery of ROP (RHO of plants) signalling components localizing to the cortical division zone seems to support this notion (Zuo *et al.*, 2014; Stöckle *et al.*, 2016). However, the question arises as to what myosin is doing in the absence of actin in the cortical division zone. In the case of myosin VIII an interaction with microtubules of the phragmoplast periphery seems to be important. Given that myosin XI does not seem to interact with MyoB1 or MyoB2 receptors during cell division it is conceivable that this motor too has a microtubule-related function. One great challenge for plant cytokinesis research is to disentangle the contributions that actin and microtubule networks provide for cell plate guidance.

The paper by Abu-Abied (2018) shows convincing evidence that the 3KO myosin mutants have both auxin-related and cell division plane orientation defects. This in itself is remarkable as auxin gradients and division plane alignment are two major patterning devices employed by multicellular land plants (Buschmann and Zachgo, 2016; Du and Scheres, 2018). But it is perfectly possible that these are fully independent capacities of myosin XI: the auxin-related defects seen in myosin 3KO plants may result from dampened cytoplasmic streaming and concurrent effects on PIN1 transporter trafficking while the cytokinetic phenotype of myosin 3KO plants may point towards a direct role of myosin XI in cell plate guidance (as discussed above). Future research can clarify whether there is cross-talk between auxin transport and cell division plane orientation in the stele cells of Arabidopsis.
